# Clinical Profile of Children With Typhoid and Paratyphoid Fever and Antimicrobial Susceptibility Testing Patterns of the Bacterial Isolates: A Cross-Sectional Study

**DOI:** 10.7759/cureus.113008

**Published:** 2026-07-20

**Authors:** Chinmay Kumar Behera, Mukesh K Jain, Anupama Singh, Abhipsa Singh, Jyoti Prakash Sahoo, Kumudini Panigrahi

**Affiliations:** 1 Pediatrics, Kalinga Institute of Medical Sciences, Bhubaneswar, IND; 2 Pharmacology, Kalinga Institute of Medical Sciences, Bhubaneswar, IND; 3 Microbiology, Kalinga Institute of Medical Sciences, Bhubaneswar, IND

**Keywords:** antibiogram pattern, antibiotic resistance trend, antimicrobial susceptibility testing, chord diagram, community-acquired diseases, enteric fever in children, length of hospitalization, paratyphoid fever, pediatric typhoid fever, salmonella infection

## Abstract

Background and objectives

Typhoid and paratyphoid fevers in children are global health concerns due to antimicrobial resistance (AMR) among their pathogens (i.e., *Salmonella typhi* and *Salmonella paratyphi*). We conducted this study to determine the symptoms of typhoid and paratyphoid fevers in children and assess the antimicrobial susceptibility testing (AST) patterns of *S. typhi* and *S. paratyphi* isolates in our hospital.

Methods

This cross-sectional study was conducted from July 2023 to June 2025 at Kalinga Institute of Medical Sciences (KIMS), Bhubaneswar, India. All pediatric patients (under 18 years) admitted during the indicated timeframe with fever were screened. The study included participants with a positive blood culture for *S. typhi* or *S. paratyphi*. Patients who reported having any additional bacteria were not included. We noted the participants’ age, sex, symptoms, and length of hospitalization. Blood samples were collected to determine AST results for the bacterial isolates. We presented our findings using an upset plot, jitter plots, and chord diagrams. We used the VITEK 2 system to perform AST. We used R software (version 4.6.1) for data analysis.

Results

Our study population comprised 328 participants (men: 171, 52.13%; women: 157, 47.87%). The median age of the participants was 8.63 (5.98-11.52) years. Typhoid and paratyphoid fevers were seen in 264 (80.49%) and 64 (19.51%) patients, respectively. The causative organisms were *S. typhi* and *S. paratyphi A*, respectively. The median hospitalization length was 7.5 (5.0-10.3) days. The most common symptoms were fever (328, 100%), followed by cough (157, 47.87%), followed by altered bowel movements (126, 38.41%), abdominal pain (89, 27.13%), coated tongue (71, 21.65%), vomiting (66, 20.12%), and loss of appetite (47, 14.33%). The *S. typhi* isolates demonstrated the highest sensitivity to chloramphenicol (261, 98.86%), followed by azithromycin (256, 96.97%), imipenem (241, 91.29%), cotrimoxazole (251, 95.08%), and meropenem (249, 94.32%). Levofloxacin (177, 67.05%) was the most resistant drug, followed by ciprofloxacin (161, 60.98%) and ofloxacin (127, 48.11%) for the 264 *S. typhi* isolates. None of the isolates showed resistance to azithromycin or chloramphenicol. The *S. paratyphi A* isolates demonstrated maximum sensitivity towards meropenem (57, 89.06%), followed by imipenem (55, 85.94%), chloramphenicol (54, 84.38%), and cotrimoxazole (51, 79.69%). Ciprofloxacin (47, 73.44%), followed by ofloxacin (42, 65.63%) and levofloxacin (41, 64.06%), were the most resistant drugs among the 64 *S. paratyphi A* isolates.

Conclusion

Most participants had typhoid fever. *S. paratyphi A* was the causative organism of paratyphoid fever in the remaining 64 participants. Fever, cough, abnormal bowel motions, abdominal pain, coated tongue, vomiting, and appetite loss were the most prevalent symptoms. For the *S. typhi* isolates, azithromycin, cotrimoxazole, imipenem, meropenem, and chloramphenicol were the most effective treatment options. Similarly, the best therapies for the *S. paratyphi A* isolates were imipenem, meropenem, cotrimoxazole, and chloramphenicol. Both isolates exhibited the highest level of resistance to quinolones, including levofloxacin, ciprofloxacin, and ofloxacin.

## Introduction

Enteric fever includes typhoid (caused by *Salmonella typhi*) and paratyphoid fever (caused by *Salmonella paratyphi A*, *B*, and *C*) [1,2]. The most common symptoms of enteric fever in children are fever and abdominal pain [2,3]. The non-specific symptoms include cough, constipation, diarrhea, nausea, vomiting, a coated tongue, and loss of appetite [2-4]. The age-specific and geographic incidence and mortality of typhoid fever in India remain uncertain despite several recent studies [5-8]. A population-based, multicentric study called Surveillance for Enteric Fever in India (SEFI) was conducted from 2017 to 2020 to determine the incidence of typhoid fever in children aged six months to 14 years at six hospital-based and four community-based surveillance sites [5]. Hospital-based surveillance sites had an incidence rate of 35-1173 cases per 100,000 person-years, according to the SEFI study. At six hospital-based monitoring sites, the incidence among people aged 15 and above ranged from 108 to 970 cases per 100,000 person-years [9,10]. Enteric fever is more prevalent in low- and middle-income countries, particularly in South Asia and sub-Saharan Africa [6,11]. A meta-analysis by John et al. reported that the population-based pooled incidence of paratyphoid and typhoid in India was 105 and 377 per 100,000 person-years, respectively [12]. The majority of cases were seen among children aged two to four years [11,12].

The main treatment for paratyphoid and typhoid fevers is antibiotics [10]. The increasing prevalence of antibiotic-resistant bacteria has compromised the efficacy of antimicrobial therapy [13-15]. India reflects the global concern over antimicrobial resistance (AMR) [16-18]. Quinolone resistance-determining region (QRDR) mutations in *S. typhi* have been detected in South Asia [14,19]. A major outbreak of *S. typhi* with plasmid-mediated resistance to third-generation cephalosporins and fluoroquinolones was discovered in Pakistan in 2016 and was designated as extensively drug-resistant (XDR) [20]. Sajib et al. identified a single-nucleotide polymorphism in the AcrB efflux pump that confers azithromycin resistance in *S. typhi* [21].

Some recent studies reported the Indian subcontinent as one of the major contributors to global incidences of typhoid and paratyphoid fever [8,10]. Children aged below 15 years comprised the majority of patients [8,10]. Therefore, we planned this cross-sectional study to determine the clinical symptoms of typhoid and paratyphoid fevers among the pediatric patients admitted to our hospital. We also evaluated the antimicrobial susceptibility testing (AST) data of *S. typhi* and *S. paratyphi* isolates detected in the study population.

## Materials and methods

This cross-sectional study examined the clinical features of typhoid and paratyphoid fevers among pediatric patients admitted to Kalinga Institute of Medical Sciences (KIMS) between July 2023 and June 2025, as well as the AST patterns of their bacterial isolates. We got the ethical clearance from the Institutional Ethics Committee (KIIT/KIMS/IEC/1363/2023, dated July 27, 2023) before starting the study.

Study criteria

We screened all pediatric patients (aged under 18 years) admitted during the above-mentioned time period with fever. Participants with a positive blood culture for *S. typhi* or *S. paratyphi* were included in the study. Patients with positive reports of other bacteria were excluded. We also excluded all referred cases.

Study procedure

The study was conducted using non-repeat clinically relevant isolates of *S. typhi* and *S. paratyphi* obtained from the participants. We recorded participants’ age, gender, symptom duration, and hospitalization length. We used the Kuppuswamy classification to classify the participants’ socioeconomic class [22]. The blood samples were collected in BacT/Alert (BioMérieux, Marcy-l'Étoile, France) broth bottles and were incubated in the BacT/Alert 3D incubator (BioMérieux, Marcy-l'Étoile, France). The *S. typhi* and *S. paratyphi* isolates were detected using the GN card in the VITEK 2 automated system (BioMérieux, Marcy-l'Étoile, France). Their AST findings were checked through the VITEK AST card N-405. We performed the azithromycin E test for *S. typhi* and a chloramphenicol disk diffusion test for both *S. typhi* and *S. paratyphi* isolates. Those AST results were assessed using the 2024 cut-off values set by the Clinical and Laboratory Standards Institute (CLSI) [23].

Statistical analysis

We adopted non-probability convenience sampling in this cross-sectional study. The normality of continuous data (e.g., age and durations of symptoms and hospitalization) was evaluated by the Kolmogorov-Smirnov test. Continuous data were expressed as median and interquartile range (IQR). Categorical data were expressed as numbers and percentages. The symptoms and AST findings were analyzed with descriptive statistics. The participants' distribution was shown via an upset plot. The symptoms were shown through jitter plots. Individual symptoms were highlighted with different colors. The AST findings were portrayed through chord diagrams. We used R (R Foundation for Statistical Computing, Vienna, Austria) version 4.6.1 [24] for data analysis and plot generation.

## Results

In this cross-sectional study, we assessed the 328 pediatric patients admitted to KIMS between July 2023 and June 2025 with typhoid and paratyphoid fever and their culture reports for AST findings. Their sociodemographic parameters are shown in Table 1. The median age was 8.63 (5.98-11.52) years. There were 171 (52.13%) male and 157 (47.87%) female patients in our study. Of them, 264 (80.49%) patients had typhoid fever and 64 (19.51%) had paratyphoid fever. The causative organisms in our study for typhoid and paratyphoid fevers were *S. typhi* and *S. paratyphi A*, respectively. Among the study participants, 110 (33.54%) patients belonged to the lower socioeconomic class, and 218 (66.46%) were from the lower-middle socioeconomic class. The median highest body temperature was 102.8°F (102.0-103.2°F). The median duration of hospital stay was 7.5 (5.0-10.3) days. Eighty-eight (26.83%) patients were hospitalized for >10 days. All patients were discharged, and there was no mortality.

**Table 1:** Demographic characteristics of the study participants The continuous and categorical variables are shown as medians with IQRs and frequencies with percentages, respectively. IQR: interquartile range.

Parameter	Value
Total participants	328
Age (years)	8.63 (5.98-11.52)
Gender	
Male	171 (52.13%)
Female	157 (47.87%)
Typhoid fever	264 (80.49%)
Paratyphoid fever	64 (19.51%)
Socioeconomic class	
Lower	110 (33.54%)
Lower middle	218 (66.46%)
Highest temperature (in °F)	102.8 (102.0-103.2)
Duration of hospitalization (days)	7.5 (5.0-10.3)
Hospital stay >10 days	88 (26.83%)

**Figure 1:**
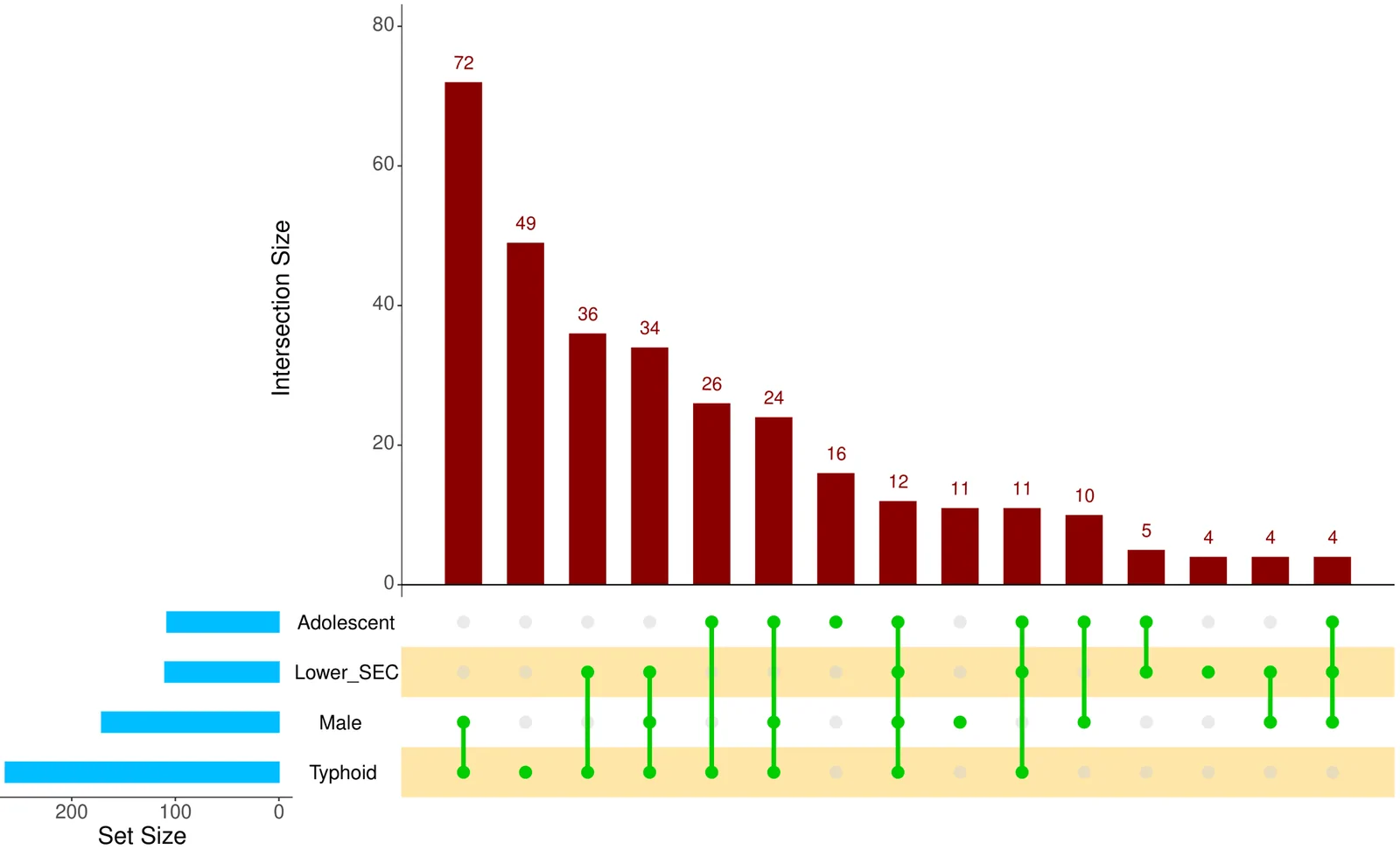
Distribution of the study population The distribution of the study population is shown in an upset plot. The horizontal bar plot (blue) shows the participants in the four groups (i.e., participants with typhoid fever, males, participants of lower socioeconomic class, and adolescent participants). The matrix (green) shows the intersections through the connecting lines and the highlighted dots. The vertical bar plot (red) shows the number of participants in those intersections. Both bar plots are arranged in decreasing order. SEC: socioeconomic class.

Figure 1 demonstrates the distribution of the study population using an upset plot. It shows two sets of bar plots (one vertical and one horizontal) and a matrix that illustrates the intersections of the parameters of the horizontal bar plot (i.e., participants with typhoid fever, men, participants of lower socioeconomic class, and adolescent participants). Both bar plots were displayed in decreasing order. The 72 (21.95%) male and 49 (14.94%) female patients aged <10 years, belonging to the lower-middle socioeconomic class, had typhoid fever.

Analysis of the symptoms

**Figure 2:**
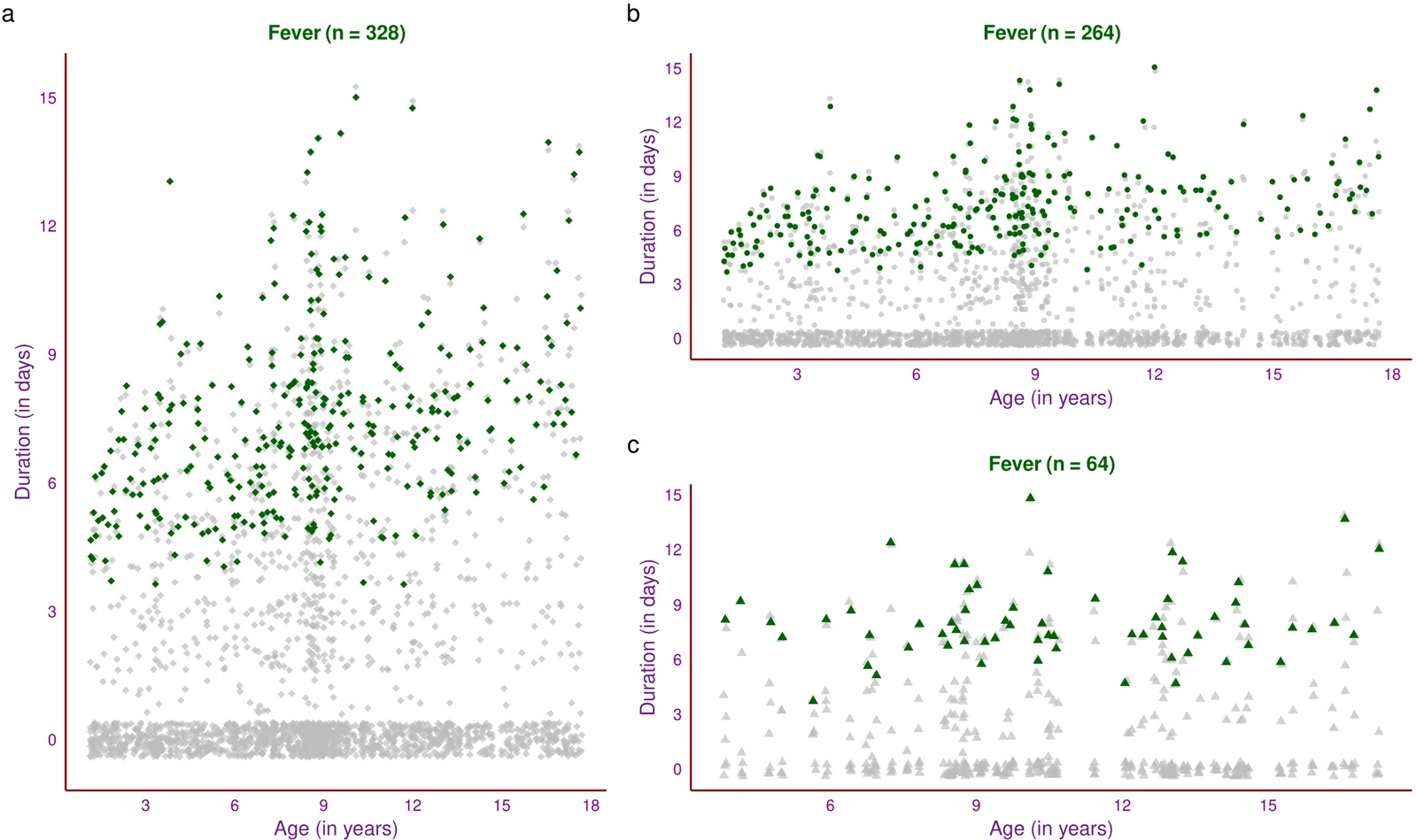
Fever duration among the study participants The jitter plots show the duration of fever among the study population. The x- and y-axes show the participants’ age (in years) and duration of fever (in days), respectively. Panels a-c show the fever durations among all participants and those with typhoid and paratyphoid A fever, respectively.

The jitter plots in Figure 2 demonstrate the fever durations of study participants. Figure 2a shows the fever duration of all participants (n=328). The median duration of fever in the study population was seven (six to eight) days. Figure 2b shows the fever duration of participants with typhoid fever (n=264). Their median fever duration was seven (six to eight) days. Figure 2c shows the fever duration of participants with paratyphoid fever (n=64). Their median fever duration was eight (seven to nine) days.

**Figure 3:**
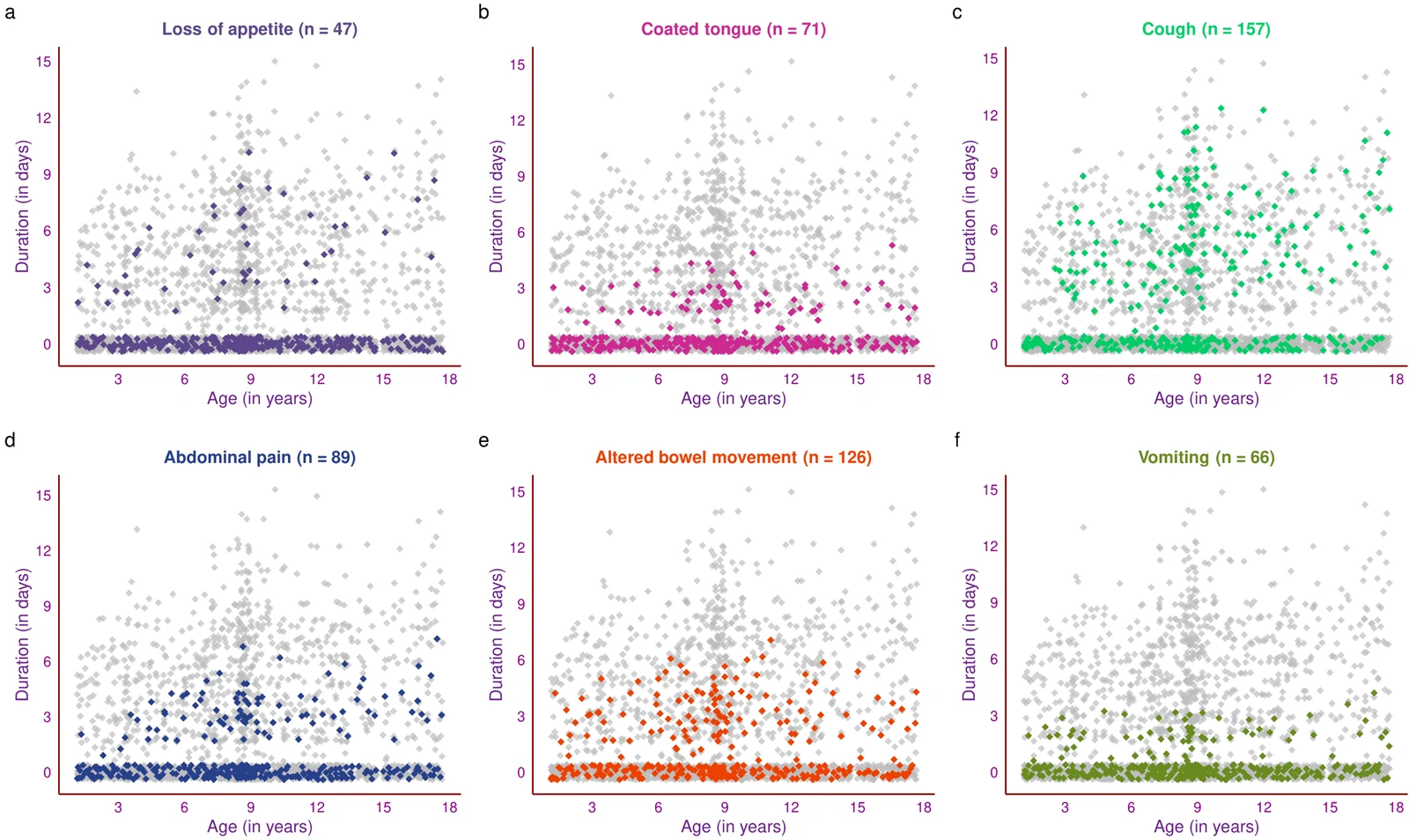
Symptoms other than fever among the study participants (n=328) The jitter plots in panels a-f show the durations of loss of appetite, coated tongue, cough, abdominal pain, altered bowel movements, and vomiting among the study population (n=328), respectively. The x- and y-axes show the participants’ age (in years) and duration of the corresponding symptoms (in days), respectively.

The jitter plots in Figure 3 illustrate the durations of symptoms other than fever of study participants (n=328). The symptoms (i.e., loss of appetite, coated tongue, cough, abdominal pain, altered bowel movements, and vomiting) were highlighted in different colors in Figures 3a-f. The decreasing order of their presenting symptoms was as follows: cough (157, 47.87%), followed by altered bowel movements (126, 38.41%), abdominal pain (89, 27.13%), coated tongue (71, 21.65%), vomiting (66, 20.12%), and loss of appetite (47, 14.33%).

**Figure 4:**
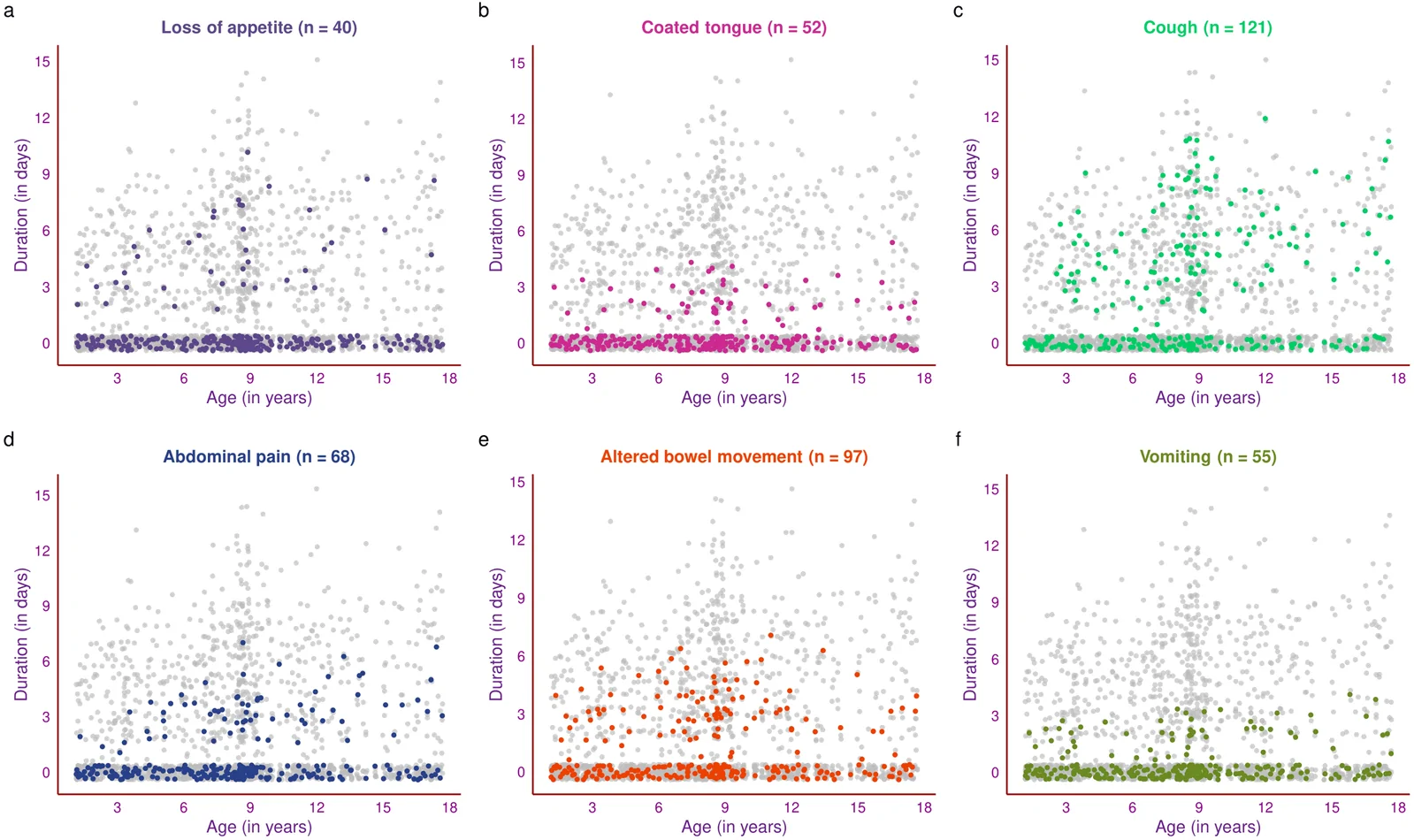
Symptoms other than fever among the participants with typhoid fever (n=264) The jitter plots in panels a-f show the durations of loss of appetite, coated tongue, cough, abdominal pain, altered bowel movements, and vomiting among the participants with typhoid fever (n=264), respectively. The x- and y-axes show the participants’ age (in years) and duration of the corresponding symptoms (in days), respectively.

The jitter plots in Figure 4 illustrate the durations of symptoms other than fever among participants with typhoid fever (n=264). The symptoms (i.e., loss of appetite, coated tongue, cough, abdominal pain, altered bowel movements, and vomiting) were highlighted in different colors in Figures 4a-f. The decreasing order of their presenting symptoms was as follows: cough (121, 45.83%), followed by altered bowel movements (97, 36.74%), abdominal pain (68, 25.76%), vomiting (55, 20.83%), coated tongue (52, 19.70%), and loss of appetite (40, 15.15%).

**Figure 5:**
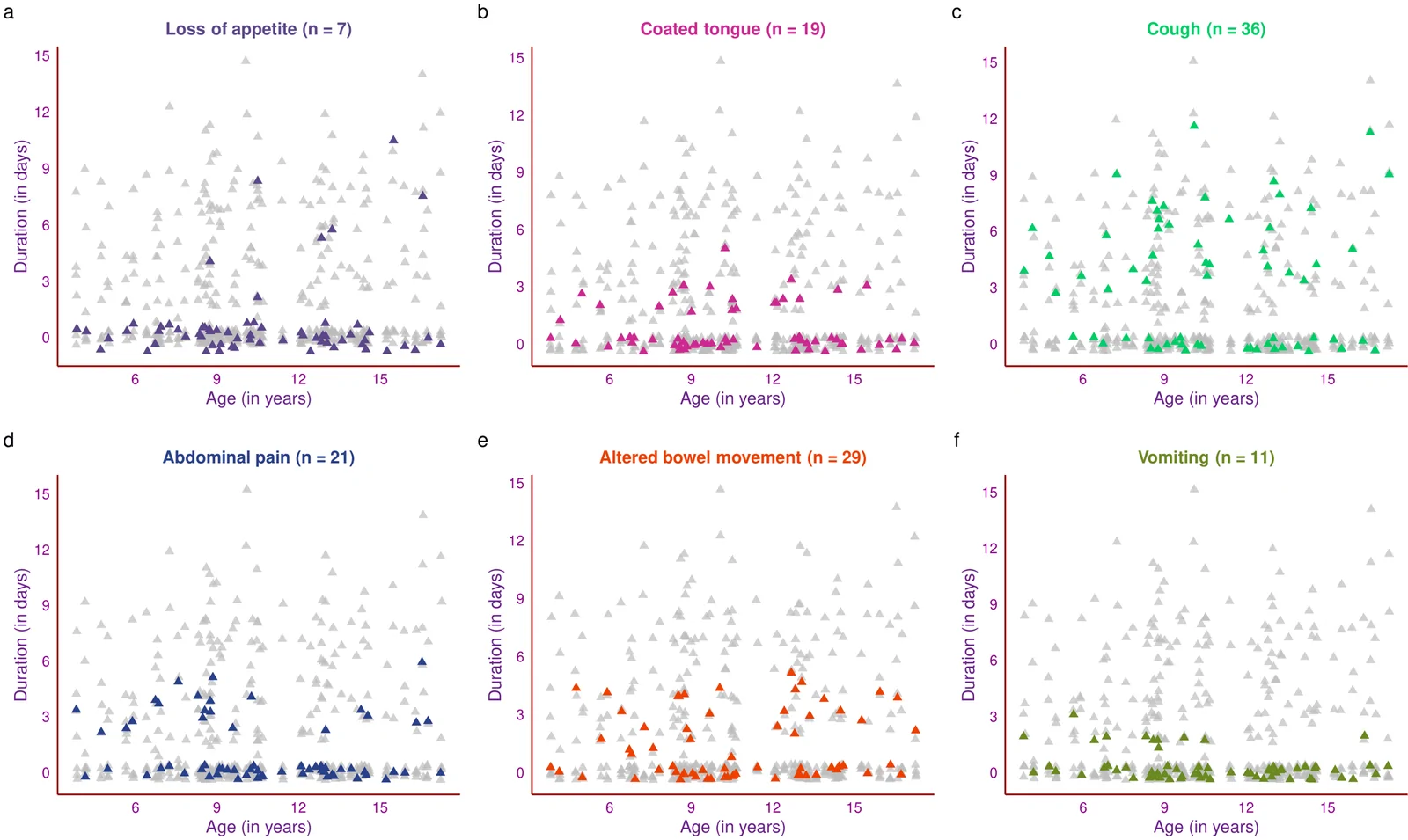
Symptoms other than fever among the participants with paratyphoid fever (n=64) The jitter plots in panels a-f show the durations of loss of appetite, coated tongue, cough, abdominal pain, altered bowel movements, and vomiting among the participants with paratyphoid fever (n=64), respectively. The x- and y-axes show the participants’ age (in years) and duration of the corresponding symptoms (in days), respectively.

The jitter plots in Figure 5 illustrate the durations of symptoms other than fever among participants with paratyphoid fever (n=64). The symptoms (i.e., loss of appetite, coated tongue, cough, abdominal pain, altered bowel movements, and vomiting) were highlighted in different colors in Figures 5a-f. The decreasing order of their presenting symptoms was as follows: cough (36, 56.25%), followed by altered bowel movements (29, 45.31%), abdominal pain (21, 32.81%), coated tongue (19, 29.69%), vomiting (11, 17.19%), and loss of appetite (7, 10.94%).

Analysis of the AST findings

**Figure 6:**
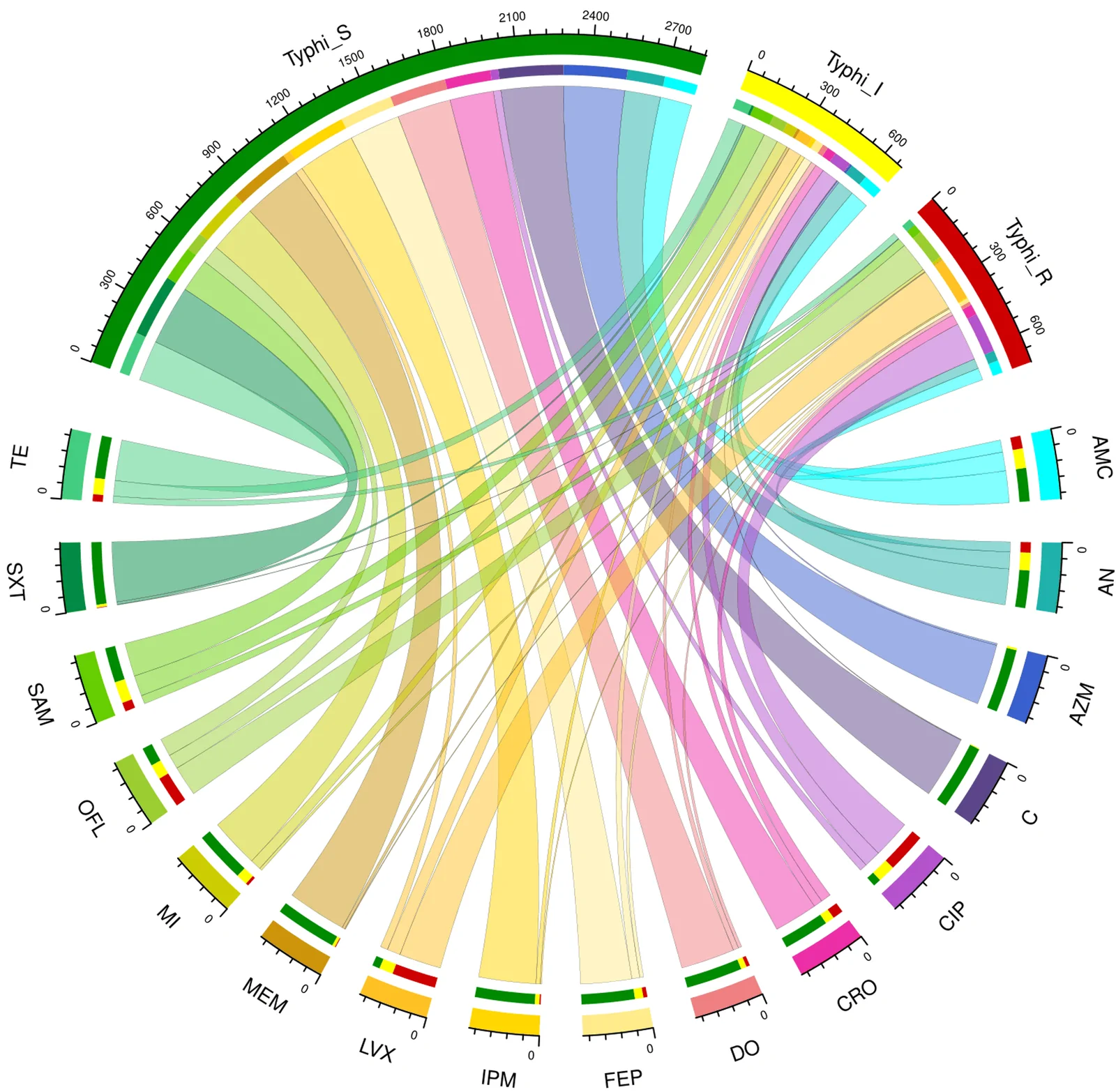
AST findings of Salmonella typhi isolates (n=264) The upper and lower sections indicate three antimicrobial susceptibility categories (S: sensitive, I: intermediate, and R: resistant) for *Salmonella typhi* specimens from participants (n=264) and 16 antimicrobials (shown in different colors). The band widths represent the number of *S. typhi* specimens and their AST profiles for the 16 drugs. AST: antimicrobial susceptibility testing, AMC: amoxicillin-clavulanic acid, AN: amikacin, AZM: azithromycin, C: chloramphenicol, CIP: ciprofloxacin, CRO: ceftriaxone, DO: doxycycline, FEP: cefepime, IPM: imipenem, LVX: levofloxacin, MEM: meropenem, MI: minocycline, OFL: ofloxacin, SAM: ampicillin-sulbactam, SXT: cotrimoxazole, TE: tetracycline.

Figure 6 and Table 2 illustrate the AST results for *S. typhi* isolates among participants with typhoid fever (n=264). Figure 6 demonstrates the susceptibility of the 264 *S. typhi* isolates and 16 antimicrobials in the upper and lower sections of a chord diagram, respectively. The maximum sensitivity was seen towards chloramphenicol (261, 98.86%), followed by azithromycin (256, 96.97%), cotrimoxazole (251, 95.08%), meropenem (249, 94.32%), and imipenem (241, 91.29%). The resistance was the highest against levofloxacin (177, 67.05%), followed by ciprofloxacin (161, 60.98%) and ofloxacin (127, 48.11%). No isolates demonstrated resistance against chloramphenicol and azithromycin.

**Table 2:** AST findings of Salmonella typhi isolates (n=264) The AST findings are expressed as frequencies and proportions. AST: antimicrobial susceptibility testing, AMC: amoxicillin-clavulanic acid, AN: amikacin, AZM: azithromycin, C: chloramphenicol, CIP: ciprofloxacin, CRO: ceftriaxone, DO: doxycycline, FEP: cefepime, IPM: imipenem, LVX: levofloxacin, MEM: meropenem, MI: minocycline, OFL: ofloxacin, SAM: ampicillin-sulbactam, SXT: cotrimoxazole, TE: tetracycline.

Drugs	Sensitive	Intermediate	Resistant
AMC	134 (50.76%)	79 (29.92%)	51 (19.32%)
AN	151 (57.20%)	71 (26.89%)	42 (15.91%)
AZM	256 (96.97%)	8 (3.03%)	0
C	261 (98.86%)	3 (1.14%)	0
CIP	32 (12.12%)	71 (26.89%)	161 (60.98%)
CRO	184 (69.70%)	37 (14.02%)	43 (16.29%)
DO	227 (85.98%)	23 (8.71%)	14 (5.30%)
FEP	214 (81.06%)	34 (12.88%)	16 (6.06%)
IPM	241 (91.29%)	18 (6.82%)	5 (1.89%)
LVX	31 (11.74%)	56 (21.21%)	177 (67.05%)
MPM	249 (94.32%)	12 (4.55%)	3 (1.14%)
MI	207 (78.41%)	46 (17.42%)	11 (4.17%)
OFL	76 (28.79%)	61 (23.11%)	127 (48.11%)
SAM	141 (53.41%)	87 (32.95%)	36 (13.64%)
SXT	251 (95.08%)	11 (4.17%)	2 (0.76%)
TE	171 (64.77%)	65 (24.62%)	28 (10.61%)

**Figure 7:**
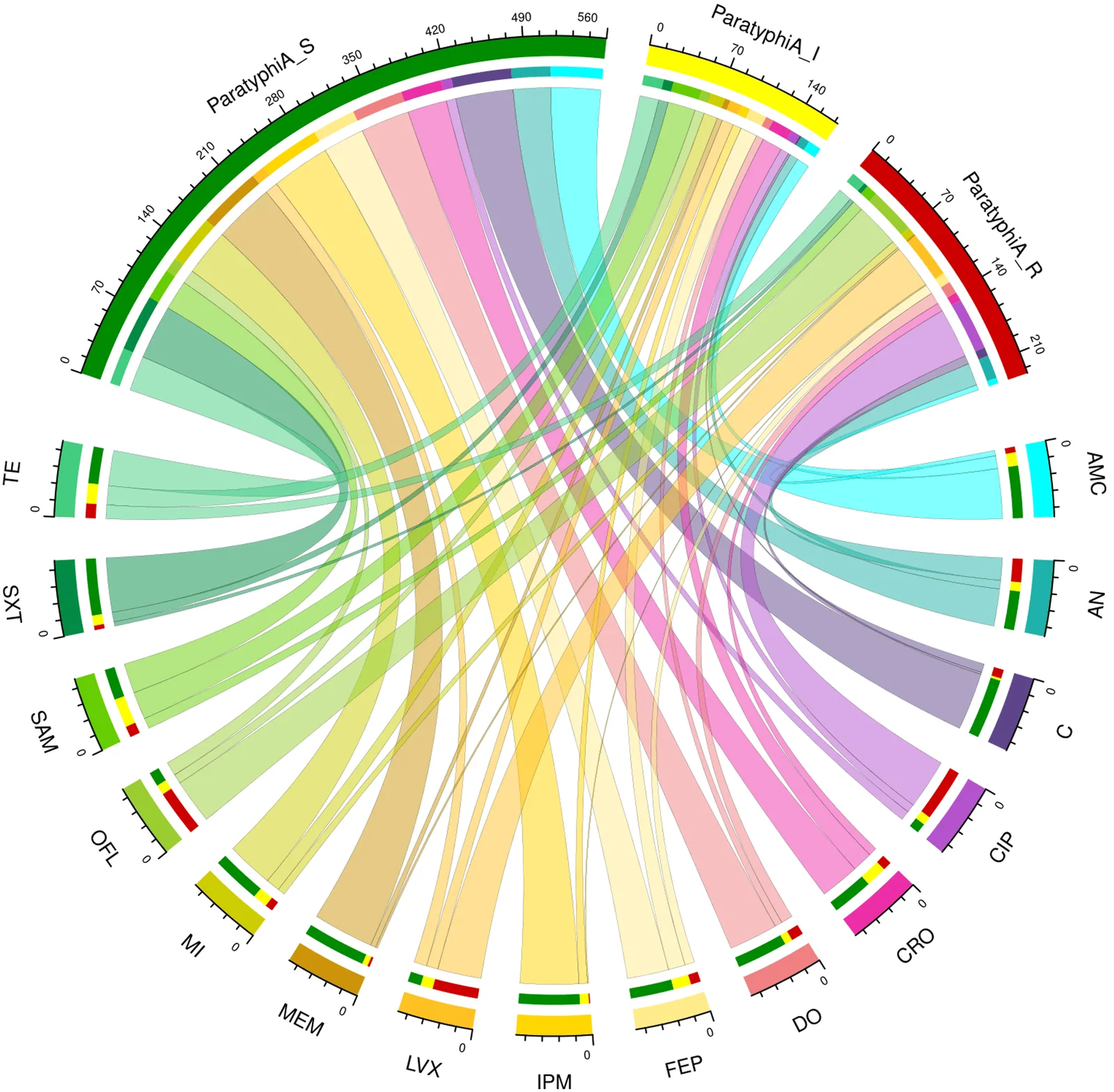
AST findings of Salmonella paratyphi A isolates (n = 64) The upper and lower sections indicate 3 antimicrobial susceptibility categories (S: sensitive, I: intermediate, and R: resistant) for *Salmonella paratyphi A* specimens from participants (n=64) and 15 antimicrobials (shown in different colors). The band widths represent the number of *S. paratyphi A* specimens and their AST profiles for the 15 drugs. AST: antimicrobial susceptibility testing, AMC: amoxicillin-clavulanic acid, AN: amikacin, C: chloramphenicol, CIP: ciprofloxacin, CRO: ceftriaxone, DO: doxycycline, FEP: cefepime, IPM: imipenem, LVX: levofloxacin, MEM: meropenem, MI: minocycline, OFL: ofloxacin, SAM: ampicillin-sulbactam, SXT: cotrimoxazole, TE: tetracycline.

Figure 7 and Table 3 illustrate the AST results for *S. paratyphi A* isolates among participants with paratyphoid fever (n=64). Figure 7 demonstrates the susceptibility of the 64 *S. paratyphi A* isolates and 15 antimicrobials in the upper and lower sections of a chord diagram, respectively. The maximum sensitivity was seen towards meropenem (57, 89.06%), followed by imipenem (55, 85.94%), chloramphenicol (54, 84.38%), and cotrimoxazole (51, 79.69%). The resistance was the highest against ciprofloxacin (47, 73.44%), followed by ofloxacin (42, 65.63%), and levofloxacin (41, 64.06%). The resistance was lowest against imipenem (1, 1.56%), followed by meropenem (2, 3.13%) and cotrimoxazole (4, 6.25%).

**Table 3:** AST findings of Salmonella paratyphi A isolates (n=64) The AST findings are expressed as frequencies and proportions. AST: antimicrobial susceptibility testing, AMC: amoxicillin-clavulanic acid, AN: amikacin, C: chloramphenicol, CIP: ciprofloxacin, CRO: ceftriaxone, DO: doxycycline, FEP: cefepime, IPM: imipenem, LVX: levofloxacin, MEM: meropenem, MI: minocycline, OFL: ofloxacin, SAM: ampicillin-sulbactam, SXT: cotrimoxazole, TE: tetracycline.

Drugs	Sensitive	Intermediate	Resistant
AMC	47 (73.44%)	12 (18.75%)	5 (7.81%)
AN	35 (54.69%)	8 (12.50%)	21 (32.81%)
C	54 (84.38%)	2 (3.13%)	8 (12.50%)
CIP	9 (14.06%)	8 (12.50%)	47 (73.44%)
CRO	37 (57.81%)	19 (29.69%)	8 (12.50%)
DO	46 (71.88%)	7 (10.94%)	11 (17.19%)
FEP	39 (60.94%)	16 (25.00%)	9 (14.06%)
IPM	55 (85.94%)	8 (12.50%)	1 (1.56%)
LVX	12 (18.75%)	11 (17.19%)	41 (64.06%)
MPM	57 (89.06%)	5 (7.81%)	2 (3.13%)
MI	44 (68.75%)	13 (20.31%)	7 (10.94%)
OFL	13 (20.31%)	9 (14.06%)	42 (65.63%)
SAM	28 (43.75%)	26 (40.63%)	10 (15.63%)
SXT	51 (79.69%)	9 (14.06%)	4 (6.25%)
TE	33 (51.56%)	18 (28.13%)	13 (20.31%)

## Discussion

The clinical features of 328 participants with typhoid and paratyphoid fevers were assessed in this study. We also evaluated the AST findings of the bacterial isolates. Fever was seen in all participants with a median duration of seven (six to eight) days. Other symptoms (in decreasing order of incidence) were cough, altered bowel movements, abdominal pain, coated tongue, vomiting, and loss of appetite. The majority (264, 80.49%) of the study population had typhoid fever due to *S. typhi*. The remaining 64 (19.51%) had paratyphoid fever due to *S. paratyphi A*. The *S. typhi* isolates were mainly sensitive to chloramphenicol, azithromycin, cotrimoxazole, meropenem, and imipenem. Similarly, the *S. paratyphi A* isolates were mainly sensitive to meropenem, imipenem, chloramphenicol, and cotrimoxazole. Both isolates showed maximum resistance towards the quinolones (e.g., levofloxacin, ciprofloxacin, and ofloxacin). Our study matched recent studies by Srinivasan et al. [4] and Dudeja et al. [11] in terms of age, gender, and clinical symptoms. Our AST findings were similar to those of Britto et al. [18] and Katiyar et al. [25].

The hot and humid climate in India leads to a scarcity of safe drinking water and an increased intake of unhealthy refreshments, such as locally made flavored drinks [4,26]. These factors can be regarded as hindrances to hygienic practices and risk factors for typhoid and paratyphoid fever [4]. Accurate and rapid diagnostics are the first step towards treating any disease, yet they often remain unavailable, inaccessible, and unaffordable in India [13,15,27]. Due to inadequate disease-reporting systems, infrequent laboratory testing, and a lack of reliable, affordable, quick diagnostic instruments, most states remain unsure of the actual enteric fever burden [13,27]. The clinical diagnosis of typhoid fever, which is frequently erroneous, is preferred over diagnostic tests in endemic and resource-limited nations [13,15,27]. Nevertheless, the presence of other acute febrile disorders renders the clinical diagnosis of typhoid relatively challenging. Ultimately, accurate laboratory testing reveals the true illness burden and initiates appropriate interventions [27].

In India, ciprofloxacin resistance began to develop in the late 1990s [18,28]. In our study, we observed the greatest resistance to quinolone antibiotics. According to a recent investigation of *S. typhi* genome sequences, antimicrobial-resistant *S. typhi* clones have multiplied and disseminated internationally in tandem with the frequent acquisition of AMR by plasmids or homoplastic mutations across many lineages [14]. All *S. typhi* lineages witnessed the independent emergence of *QRDR* gene mutations [14,19]. According to the phylogeographical research by da Silva et al. [14], these *S. typhi* clones most likely originated in India. A method for sustained vertical transmission of multidrug-resistant phenotypes is provided by the incorporation of AMR genes into the *S. typhi* chromosome [14]. Nowadays, physicians often prescribe azithromycin and ceftriaxone for the treatment of enteric fever in India [28]. The bacterial isolates in our study population were mainly sensitive to chloramphenicol, azithromycin, cotrimoxazole, meropenem, and imipenem. Nevertheless, growing AMR, disease severity, hospital burden, and mortality rate warrant monitoring of clinical features and serology, rapid and accurate diagnostics, and avoidance of the last-resort antimicrobials in therapy [28].

Strengths and limitations

We assessed two years of data on clinical symptoms of enteric fever in pediatric patients and on AST findings of bacterial isolates. The upset plot of the study population’s distribution, jitter plots of the symptoms, and chord diagrams of the AST findings illustrated our observations. There were a few limitations in our study. First, the study population was constrained by the study criteria. Hence, the symptoms and AST findings cannot be generalized. Second, our findings did not represent the Indian antibiogram. Third, we could not perform or correlate phenotypic and genotypic analyses. Fourth, our study was also limited by the lack of whole-genome sequencing (WGS)-based resistance gene screening.

## Conclusions

The majority of our study population had typhoid fever. We found only *S. paratyphi A* isolates as the causative organism of paratyphoid fever in one-fifth of our participants. The most common symptoms were fever, cough, altered bowel movements, abdominal pain, coated tongue, vomiting, and loss of appetite. Chloramphenicol, azithromycin, cotrimoxazole, meropenem, and imipenem were the most effective treatments for the *S. typhi* isolates. Similarly, meropenem, imipenem, cotrimoxazole, and chloramphenicol were the most effective treatments for the *S. paratyphi A* isolates. Both isolates demonstrated the highest resistance to quinolones, such as levofloxacin, ciprofloxacin, and ofloxacin. Increases in AMR, disease severity, healthcare costs, and fatality rates necessitate timely and precise diagnostics, clinical surveillance, and the minimization of last-line antibiotics in therapy. Therefore, we recommend prospective studies with larger sample sizes to determine the AST findings and resistance genes of *S. typhi* and *S. paratyphi* isolates.
